# Qualitative assessment of infant sleep practices and other risk factors of sudden infant death syndrome (SIDS) among mothers in Lusaka, Zambia

**DOI:** 10.1186/s12887-023-04051-9

**Published:** 2023-05-18

**Authors:** Godwin K. Osei-Poku, Lawrence Mwananyanda, Patricia A. Elliott, William B. MacLeod, Somwe Wa Somwe, Rachel C. Pieciak, Arnold Hamapa, Christopher J. Gill

**Affiliations:** 1 Department of Global Health, Boston University School of Public Health, 801 Massachusetts Avenue, Crosstown Center, 3rd Floor, Boston, MA 02118 USA; 2 Department of Community Health, Boston University School of Public Health, 801 Massachusetts Avenue, Crosstown Center, 4th Floor, Boston, MA 02118 USA; 3 Department of Pediatrics, School of Medicine, University of Zambia, Lusaka, Zambia; 4 School of Public Health, University of Zambia, Lusaka, Zambia

**Keywords:** SIDS, Sudden infant death, Bedsharing, Sleep position, FGDs, Sleep Practices

## Abstract

**Background:**

There is very little information on the beliefs and perceptions of mothers about SIDS and its related risk factors in Africa. To better understand parental decisions about infant sleep practices and other risk factors for SIDS, we conducted focus group discussions (FGDs) with mothers of infants in Lusaka, Zambia.

**Methods:**

FGDs involved 35 purposively sampled mothers aged 18–49 years. FGDs were conducted using a semi-structured interview guide in the local language, Nyanja. These were translated, transcribed verbatim into English, and then coded and analyzed using thematic analysis in NVivo 12.

**Results:**

Six FGDs were conducted with 35 mothers in April-May 2021 across two study sites. FGD Participants were generally aware of sudden unexplained infant deaths, with several describing stories of apparent SIDS in the community. The side sleeping position was preferred and perceived to be safer for the infant with most believing the supine position posed an aspiration or choking risk to the infant. Bedsharing was also preferred and perceived to be convenient for breastfeeding and monitoring of the infant. Experienced family members such as grandmothers and mothers-in-law, and health care workers were frequently cited as sources of information on infant sleep position. A heightened awareness of the infant’s sleeping environment was suggested as a mechanism to prevent SIDS and smothering.

**Conclusions:**

Decisions about bedsharing and infant sleep position were guided by maternal beliefs and perceptions about what is convenient for breastfeeding and safer for the infant. These concerns are vital to designing tailored interventions to address sleep-related sudden infant losses in Zambia. Public health campaigns with tailored messages that address these concerns are likely to be effective at ensuring optimal uptake of safe sleep recommendations.

**Supplementary Information:**

The online version contains supplementary material available at 10.1186/s12887-023-04051-9.

## Background

In high-income countries, sudden infant death syndrome (SIDS) is consistently the most common preventable cause of infant mortality. However, in Africa, very few studies have explored the burden of SIDS [1]. In the few studies that have been conducted, the SIDS rate in Africa appears to be higher than those reported in high-income countries. For instance, the SIDS rate in South Africa has been estimated to be between 3.01 and 3.70 per 1000 live births [2, 3]. In Zambia, very little is known about the burden of SIDS. In our recent postmortem surveillance study, during the review of the free-text narratives for 809 young infants who had died in the community, we found that nearly 7.4% died suddenly during sleep without any antecedent illness, with 5.2% dying of suspected SIDS,[4] suggesting that SIDS may be a significant cause of infant mortality in Zambia but is largely overlooked.

While several risk factors have been identified for SIDS, including maternal smoking and alcohol use, [5–7] sharing a bed with the infant and having infants sleep in the prone or lateral position are the most important modifiable risk factors for SIDS [8–11]. However, few prior research studies have focused on understanding the risk factors for SIDS in Africa. In the few studies, data suggest higher rates of bedsharing and prone sleeping [1]. For instance, Ibeziako et al. found a high incidence of prone sleeping and bedsharing in a cohort of infants in South Eastern Nigeria [12]. Moreover, among sudden unexpected death in infancy (SUID) (a broader, more agnostic category that incorporates both SIDS and accidental suffocation and smothering deaths) cases in South Africa, the bedsharing rate was nearly 95% [1, 13].

In our recent survey exploring the risk factors for SIDS among mothers of infants in Zambia, we too, found high rates of these risk factors. Among the 478 mothers we surveyed, most (89.5%) preferred to share a bed with the infant [14]. Two in ten mothers reported placing their infant to bed in a prone (19.9%) position, with 73.0% preferring the lateral position [14]. However, it is unclear if the high bedsharing and lateral sleeping rates are associated with SIDS as in high-income countries, highlighting the need for additional research to understand these risk factors within the unique cultural context of Zambia. Moreover, understanding the underlying motivations for these practices will be essential to designing effective interventions to address SIDS in Zambia and Africa generally.

Our initial literature review found very little information on the beliefs and perceptions of mothers about SIDS in general and about the risk factors associated with SIDS in Africa. To the best of our knowledge, no study in Zambia, or Africa more broadly, has explored the beliefs and perceptions of mothers and other caregivers about SIDS and its associated risk factors. To better understand parental decisions on infant sleep practices and other risk factors of SIDS in Zambia, we conducted focus group discussions (FGDs) with mothers of infants at Chawama and Chilenje Hospitals in Lusaka, Zambia. Through these FGDs, we aimed to explore the beliefs and perceptions of mothers and other caregivers of infants about unexplained sudden infant deaths and the risk factors associated with these deaths in Lusaka, Zambia.

## Methods

### Sampling and recruitment

We conducted six FGDs in April-May 2021 with mothers of infants under one year. We estimated, based on prior research, that a minimum of 6 to 8 focus groups would be necessary to reach thematic saturation [15]. All participants for the FGDs were recruited from postnatal clinics at the Chawama and Chilenje General Hospitals in Lusaka, Zambia. Participants were purposively identified and recruited into the study by designated nurses at each study site. Eligible participants were aged 18–49 years, cared for or were a mother of an infant aged less than one year, resident within the catchment area of each study hospital, and able to give informed consent. Consent forms were provided in both English and Nyanja. Women who opted to participate in the study were provided written informed consent and completed a pre-FGD demographic questionnaire. Demographic questionnaires did not include personal identifiers except for age and study site location. Data collectors assisted illiterate participants by verbally reading through the consent form to obtain consent.

### Data collection

Focus group discussions were conducted in private rooms at each study site. To reduce attrition and non-participation, we recruited women and received written informed consent for their participation on the same day. FGDs were moderated by one study author (AH) and a second data collector. Both are Zambian nationals with expertise and training in qualitative interviewing in Nyanja; the participants preferred language. AH facilitated the focus groups while the second data collector took notes and asked additional questions when further clarification was needed. FGDs were typically 60–90 min long, including the consent and questionnaire process. All FGDs followed a semi-structured guide and were audio-recorded. These were then translated and transcribed verbatim into English by AH. The translations were verified and deemed to accurately reflect the views of the women who took part in these discussions by the second data collector. FGDs centered on participants’ views and perceptions of sudden infant death, infant sleep practices including bedsharing, their preferred sleep position for infants, and maternal use of alcohol or tobacco during pregnancy.

### Analysis

We analyzed FGDs using inductive thematic analysis. Two study authors (GKO-P and AH) independently analyzed transcripts of the first two focus groups when the focus groups were being conducted and generated initial codes with guidance and supervision by a third author (CJG). Themes were discussed and agreed on by the authors. One author (GKO-P) then coded all the transcripts using NVivo 12,[16] a qualitative analysis software (QSR International, Melbourne, Australia) on completion of all the focus group discussions. Transcripts and codes were revised iteratively throughout the coding process as themes emerged. Our decision to conduct six focus group discussions was validated when no new themes emerged after the fourth focus group transcript. A fourth author (PAE) reviewed the final report for consistency and accuracy.

Ethical approval was provided by the ethical review boards at Boston University Medical Center and the University of Zambia Biomedical Research Ethics Committee.

## Results

### Participant characteristics

The demographic features of study participants are presented in Table 1. Thirty-five women participated in these FGDs, with six women on average in each session (range 5–7). The mean age of study participants was 27.9 years old (*std =* 6.3 years). Most (60.0%) were between 20 and 29 years old and married or staying with a partner (80.0%) at the time of the study. Educational attainment was rarely beyond secondary school, with only 17.0% reporting post-secondary school education. Infants of participants were majority female (54.3%) with a mean age of 6.4 months (*std =* 3.5 months).

**Table 1:** Population Characteristics of FGD participants

Characteristic	Total N = 35	Study site	
		Chawama N = 17	Chilenje N = 18
Maternal Characteristics			
*Maternal age, mean (std.)*	27.9 (6.3)	27.8 (6.9)	28.1 (5.9)
*Age in years, n, %*			
< 20	1 (2.9%)	1 (5.9%)	.
20–29	21 (60.0%)	10 (58.8%)	11 (61.1%)
30–39	11 (31.4%)	5 (29.4%)	6 (33.3%)
>=40	2 (5.7%)	1 (5.9%)	1 (5.6%)
*Marital status, n (%)*			
Single/Divorced/Widowed	5 (14.3%)	1 (5.9%)	4 (22.2%)
Married/Co-habiting	28 (80.0%)	16 (94.1%)	12 (66.7%)
Missing/Unknown	2 (5.7%)	.	2 (11.1%)
*Education, n (%)*			
Primary	2 (5.7%)	.	2 (11.1%)
Secondary	27 (77.1%)	16 (94.1%)	11 (61.1%)
Postsecondary	6 (17.1%)	1 (5.9%)	5 (27.8%)
Infant Characteristics			
*Infant age, mean, std.*	6.4 (3.5)	5.2 (3.2)	7.6 (3.4)
*Infants age in months, n (%)*			
0-1mo	2 (5.7%)	2 (11.8%)	.
2-4mo	13 (37.1%)	8 (47.1%)	5 (27.8%)
5-7mo	6 (17.1%)	3 (17.6%)	3 (16.7%)
8-10mo	8 (22.9%)	3 (17.6%)	5 (27.8%)
11-12mo	6 (17.1%)	1 (5.9%)	5 (27.8%)
*Infants Gender/Sex, n (%)*			
Male	14 (40.0%)	8 (47.1%)	6 (33.3%)
Female	19 (54.3%)	9 (52.9%)	10 (55.6%)
Missing/Unknown	2 (5.7%)	.	2 (11.1%)

### Themes

The main themes and subthemes that emerged from these discussions are shown in Table 2. These themes are described below with illustrative verbatim quotes. Additional quotes describing each theme are included in the supplementary document.

**Table 2:** Main themes, subthemes and codes from FGD sessions

Main theme	Sub-theme	Codes
Awareness and Knowledge of SIDS	Awareness	− Heard about SIDS-like deaths in the community
	Presumed cause of SIDS	− Suffocation from overlay/soft bedding/ sleep position − Uncertainty about cause − Baby-abandonment − Sick Child − Fall from bed − Milk aspiration
	Mechanisms to prevent SIDS	− Vigilance or monitoring of child
Sleep position	Reason for sleep position	− Prevent aspiration/vomiting − Easy for infant to breathe well − Infant sleeps well − Chosen position is stable
	Information sources	− Family and friends − Healthcare providers
Bedsharing and room sharing	Reasons for bedsharing or room sharing	− Easy to monitor child/safety concerns − Convenient for breastfeeding − Can’t afford separate bed or room for child − Helps in maternal/infant bonding
	Mechanism to prevent smothering	− Conscious of sleeping child − Sleep carefully − Protect from sleeping father
Sleep surface	Blankets and other covering	− Comfort (Spread blanket for baby to sleep) − Pillows seldom used
	Type of mattress	− Preference for soft mattress
Bundling with blankets	Reason for bundling	− Keep infant warm
Parental alcohol use	Reasons for alcohol intake	− Pregnancy related cravings − Perception that child will be fat and beautiful − Underestimate harms of alcohol
	Other women drink	− Know other women in community who drank while pregnant
Parental smoking	Aware of harms of tobacco	− Destroys lungs or chest − Possible neonatal nicotine addiction − Can cause cancer − Likely cause SIDS − Stillbirth
	Other tobacco	− Feel good − Warms body (Sexual stimulant) − Tightens vagina − Cause cancer
	Other women smoke	− Know other women in community who smoke

### Maternal awareness and knowledge of SIDS

FGD participants were generally aware of unexplained sudden infant deaths. Although none of the participants had experienced a previous sudden infant loss, they had heard stories involving neighbors or other women in the community who had lost an otherwise healthy infant during sleep.

*“I heard of it. Both parents were medical personnel. They are saying they put the baby to bed at night, and by the time they checked on the baby in the early hours of the day, they just found the baby dead” (P2, Chawama 2)*

When asked what caused these deaths, mothers often cited suffocation resulting from smothering or soft bedding such as blankets and the infant’s sleep position.

*“I heard they slept on the baby. You know, those who drink beer they have a baby they sleep on the baby; they can’t even hear the baby crying. They wake up in the morning, they find the child is dead” (P1, Chawama-1)*

*“I just heard of the baby who died in the bus. They had taken the baby for [BCG], they covered the baby with blankets, so it seems that was too much for the baby that it caused suffocation. When the mother got home to uncover the baby only to find the baby was dead. So, I think it was due to blankets that the baby suffocated” (P6, Chilenje-3)*

*“I just heard. There was a friend we went to the same school. She said that my sister had put the baby to sleep while facing down so then the baby was not breathing fine that is how in the morning the baby was found dead” (P2, Chilenje-2)*

However, other participants were uncertain about what caused these deaths, indicating that they did not know or were not told what had caused the infant’s death.

*“.. My sister-in-law had put the child to bed thereafter they just found the child dead.*

*I: what do you think caused the death of the child?*

*I don’t know” (P1, Chawama-2)*

### Maternal beliefs and perceptions on sleep position

Most participants preferred the side and prone sleeping positions for their infants. Several indicated that these positions were safer since they prevented the infant from accidentally aspirating their vomitus during sleep. The side position was also believed to allow infants to breathe well during sleep. In contrast, the prone position was perceived to be stable, allowing the infant to sleep for extended periods. The supine position was seldom preferred since this was deemed to pose an aspiration or choking risk to the infant.

*“The reason why we place them on the sides is that even when they vomit, things will drop on the sides, but when facing up, the vomit might go into the nose, they fail to breath that is when you now find the child dead” (P4, Chawama-2)*

*“I think putting the baby on the sides at night is better because even breathing is easier….” (P5, Chilenje-3)*

*“I put them facing down. That is when they sleep well, not facing the sides. You find they turn on their own…... they think as though they are on the back because on the back, they sleep a lot so if you keep them that way, they take a long sleep” (P2, Chawama-1)*

Several participants indicated that their primary sources of information on sleep position were their mother and other experienced family members, including aunties, mothers-in-law, and grandmothers.

*“My grandma, mother-in-law and my mum they have more experience than we do because they raised us, so anything they say must be important yes” (P6, Chilenje-3)*

In addition, healthcare workers were also cited as sources of information on these sleep practices. Most indicated that they received advice from healthcare workers, such as nurses at postnatal clinics, to place the infant on the side position to sleep.

*“At the clinic when you come for first injection for BCG, they say that the best position is for the sides” (P4, Chawama-3)*

*“Me I was taught here at the hospital before and after having this baby because is the first.” (P3, Chilenje-1)*

#### Maternal beliefs and perceptions on bedsharing and room sharing

Several mothers in this study preferred sleeping in the same bed and room with the infant. Most believed that by doing so, it is easier to monitor the child and breastfeed without interrupting their sleep.

*“We sleep with the baby on the same bed because I am more comfortable when he is with me than with anyone. More probably they won’t give much attention as I do, and feeding is easier, you feed without even moving around the room. It disturbs the baby, so it’s easier when the baby is next to you” (P5, Chilenje-3)*

*“As for me, I can’t manage what the Europeans do. The baby is left in the other room, then you sleep elsewhere, maybe that side the blanket covers the baby. I can’t accept, I want to sleep with the baby closely” (P2, Chawama-1)*

Bedsharing was also thought to enhance bonding between mother and infant.

*“We share the same bed with the baby, and it is important I think, somehow it helps in bonding before the child is 2 years. You build that closeness of always being safe when your mom is around so for me, I think it is very important for the child to sleep with you” (P5, Chilenje-3)*

Mothers’ inability to afford a separate room or bed for the infant due to their socioeconomic circumstances was also cited as a reason for bedsharing.

*“……. But what makes us to sleep with them, we don’t have money to get another bed, but when they grow, you can let them sleep alone” (P1, Chawama-3)*

*“Some the baby sleeps alone in the baby cot but if you don’t have better, you sleep with the baby together” (P1, Chilenje-2)*

### Maternal perspectives on sleep surface and bundling

Several participants preferred a soft sleep surface for the infant, indicating that a soft sleep surface was more comfortable and appropriate for babies whose bodies were still soft. Most would even spread blankets on the bed or floor to provide a softer sleep surface for the infant.

*“.. They need to sleep where it is soft because even big people can’t sleep where it is hard. Our bodies pain, what more for the baby or you find that you put them where it is hard, even the bed leaves a mark on the baby (laughs). You find you carry the baby; she is crying because the body is aching” (P4, Chawama-2)*

*“It should be a soft surface not something hard because the baby is not yet how do I say it okay, something soft comfortable for the baby” (P1, Chilenje-3)*

Several participants reported bundling their babies with at least two blankets during sleep. In cold weather especially, mothers preferred to bundle their infant with blankets to keep the baby warm, often following prior advice from health care workers at the clinic. Concerns about hypothermia leading to cough, pneumonia, or even death also guided their decisions about bundling.

*“.. we need to be using blankets on the child because the child needs to be where it is warm so that they don’t get a cough or pneumonia” (P5, Chawama-1)*

*“… yes, you put a baby blanket, then you add yours on top maybe the baby blanket is not enough, so you add yours to avoid the baby from freezing” (P5, Chilenje-1)*

### Strategies to prevent suspected SIDS and smothering deaths

Participants were concerned about their infant dying suddenly during sleep. Several expressed that being vigilant was the best strategy to prevent such deaths. Most suggested frequently checking on the infant when they are sleeping, rarely leaving the infant to sleep alone, and being alert to the presence of the infant in bed as some strategies to reduce SIDS and smothering deaths.

*“.. when sleeping, I don’t have to fall into deep sleep I should be checking on the baby. Even breastfeeding, like how I feed my child, I don’t do it while sleeping, I wake up and sit or when I wake up, I have to see that the baby is safe. After doing house chores I can’t stay for an hour or 30 minutes short I go and see how the child is and when [he/she] sleeps for too long I put my child on my back when I feel tired, I take the baby back to the bed”. (P6, Chawama-3)*

*“…. The mind is alert. We know that there is the baby here [in same bed]. When the father is on this side, you know that the baby is in the middle, so when turning, you are careful. The mind is always alert” (P7, Chilenje-2)*

### Maternal perspectives on alcohol consumption and smoking during pregnancy

Maternal use of tobacco and alcohol during pregnancy was generally low among study participants. All participants reported no tobacco use during pregnancy, citing possible neonatal nicotine addiction, stillbirth, and even unexplained sudden infant death as reasons for choosing not to smoke during pregnancy.

*“…It can kill the child because they fail to breathe. That heat and smoke which goes inside affects the child. Some are born with cough, [when] they are even sick when you get medicine from the clinic they can’t help because the baby is addicted to smoke, so when you smoke in their presence that is when they feel better because they are addicted with that smoke” (P1, Chawama-1)*

*“There are others who smoke even when pregnant, so you find that they give birth, in the chest for adults they become destroyed what more the unborn baby. So, you give birth, then the baby dies in sleep” (P3, Chilenje-3)*

On the other hand, a few participants reported using alcohol during pregnancy, indicating that their use of alcohol was due to severe pregnancy-related cravings and the endorsed belief that alcohol will make their babies fair and beautiful at birth.

*“… on my side I drank alcohol on this child because I had that lust, the thirst for beer, I used to tell the father that please buy me that beer which they call score, I used to take that one, I drink I go to bed. On my side I drank, I can’t say I didn’t, I did” (P4, Chawama-1)*

*“I know someone who was even telling me saying, now that you are pregnant, you want to have a fair baby, you be taking Lusaka beer mix with sour milk. So, I think I will say that’s what I was told but I never take, I would just joke”* (P1, Chilenje-3)

Some mothers also reported knowing other women in the community who used other tobacco products while pregnant. A local tobacco product (nsunko) was often cited. Nsunko was perceived to make women “feel good” and was viewed as a sexual stimulant.

*“There are many they smoke nsunko (other tobacco), they put underneath the tongue you find they are walking it’s just there, now I ask them, now smoking nsunko (Sniffing tobacco) how do you feel, they say, they feel good” (P2, Chawama-1)*

*“Some say it warms the body for women others say they make you feel high and good that is what they say” (P1, Chilenje-3)*

## Discussion

Responses in these FGDs provide evidence that sudden infant losses are well recognized in these Zambian communities [14]. Several study participants indicated they had heard stories from neighbors or other women in the community of infants who were otherwise well and died during sleep. Most found a link between these deaths and suffocation, while others were uncertain of the cause of such deaths. Our study participants also generally believed they could prevent these apparent SIDS deaths by being more vigilant and keeping an eye on the child. In almost all instances, our study participants shared the same room with the infant, believing that this afforded them the best opportunity to monitor the child and protect them from suffocation and other harm. This belief also led to several study participants sharing the same bed with the infant. Bedsharing was perceived to provide a more convenient way to breastfeed and to monitor or keep the child safe, consistent with findings from prior qualitative studies among African American mothers in the U.S. [17, 18].

There were also socio-economic reasons for mothers in this study sharing a bed with the infant; at least one participant indicated that they “don’t have better”. In a low-resource country such as Zambia, this finding is unsurprising. Moreover, the practice of keeping an infant in a separate room was perceived to be a European practice. However, whether sharing the same bed with the infant poses an increased SIDS risk in these communities is unclear. The American Academy of Pediatrics recommends that infants do not sleep on the same sleep surface as adults to prevent SIDS [10]. However, not all experts agree that all bedsharing is unsafe for the infant [19]. In this population, the convenience of breastfeeding and the need to keep the infant safe motivated the need to bedshare. Strategies that make bedsharing and breastfeeding safer may be worthwhile in these Zambian communities.

Most mothers in our study also believed that the infant would aspirate or vomit when placed to sleep in the supine position despite evidence to the contrary [20, 21]. Instead, they preferred the lateral or side position, believing it to be safer for the infant. A soft sleep surface was also clearly preferred, although previous research has found a strong association between sleeping on a soft surface and an increased risk of SIDS [8]. Moreover, motivated by a desire to keep the infant warm, most mothers would bundle the infant with blankets and clothing during sleep, consistent with findings from prior qualitative research among Black parents in the U.S [22]. Our findings suggest that preventing respiratory infections such as pneumonia likely takes precedence over preventing SIDS in cultures where such infections are prevalent. For instance, Cronin et al. showed that thermal care practices differed across cultures – while white British mothers were concerned about overheating causing SIDS, South-Asian mothers were concerned about cold causing infections such as pneumonia. [23]. These concerns should be addressed in any SIDS interventions or programs.

In these focus group discussions, it became apparent that healthcare workers offered limited guidance on safe sleep practices. In some of our discussions, healthcare workers were credited for advising mothers to place the infant to sleep in the lateral or side position, contrary to most safe sleep recommendations. Mothers also often relied on their mothers and other family members for guidance on the best position to place the infant to sleep. Our findings on these sleep practices suggest the need for broader consultations with stakeholders, including the Ministry of Health, and a system-wide collaboration between the government and its development partners to improve healthcare worker education on safe sleep recommendations and identify low-cost interventions that could make the infants sleeping environment safer.

Discussions in the focus groups revealed that mothers were generally aware of the harms of tobacco and alcohol to the mother and unborn child. However, participants indicated they knew several women in the community who drank alcohol or used other tobacco products while pregnant. Martinelli et al. similarly found that Pregnancy-related cravings [24] motivated the use of alcohol during pregnancy in a qualitative study in Brazil. The desire to give birth to fairer, more beautiful children as a reason for consuming alcohol during pregnancy was a surprising finding requiring better education on the harms of alcohol. Moreover, pregnant women’s use of Nsunko (a local tobacco product) could inadvertently put the unborn child at risk of SIDS. The link between nicotine in tobacco and SIDS has long been established [7, 25]. Nicotine is thought to affect the arousal response in neonates [26]. Due to the local nature of Nsunko, we are unable to predict how much nicotine is in this product and its effect on an infant’s SIDS risk. Additional research is thus needed to determine the effect of these local tobacco products on an infant’s SIDS risk in Zambia.

### Strengths and limitations

To the best of our knowledge, this is the first qualitative study on the risk factors of SIDS in Zambia and Africa, more broadly. By using this study design, we have provided some context to the unsafe sleep practices we found in our earlier survey,[14] affording us a deeper understanding of maternal behaviors that may or may not put their infants at an increased or decreased risk for SIDS. Additionally, our study triangulated very well. Our findings on infant sleep practices were complementary across surveys and FGDs. Thus, we used different data collection streams to arrive at similar conclusions.

However, this study has limitations. It was a hospital-based study in two hospitals in an urban community. We did not represent the views of women in rural areas whose beliefs and perceptions on these sleep practices may differ from those expressed here. Recall, interviewer, and translation bias are also likely limitations of this study. We minimized recall bias by recruiting mothers currently caring for an infant. We also minimized interviewer bias by going into this research with no preconceived ideas of what to find. We allowed themes to emerge inductively during the study.

## Conclusions

Our findings have important implications for public health. Safe sleep campaigns have been shown to be highly effective at reducing all-cause infant mortality in other countries, but such gains can only be achieved if the recommendations are accepted and acted upon by mothers. In Zambia, current sleep practices are based on strongly held cultural beliefs and are also encouraged by the medical profession. Therefore, we suspect that implementing such campaigns in Zambia will require a deeper understanding of the beliefs and perceptions of mothers and care givers about an infant’s sleep environment, data which this study provides. In our opinion, any behavioral change campaigns that result would benefit from a high degree of community input and sensitization, ideally guided by a community-based participatory research strategy to generate evidence that is not just robust but that will be accepted by the community.

## Electronic supplementary material

Below is the link to the electronic supplementary material.

Supplementary Material 1

## Data Availability

The datasets used and/or analyzed during the current study are available from the corresponding author on reasonable request.

## List of abbreviations

SIDS: Sudden Infant Death Syndrome
SUID: Sudden Unexpected Infant Death
US: United States
BCG: Bacille Calmette-Guérin Vaccine
