# Supplementary material for: Qualitative assessment of infant sleep practices and other risk factors of sudden infant death syndrome (SIDS) among mothers in Lusaka, Zambia
Source: BMC Pediatr. 2023 May 18;23:245. doi: 10.1186/s12887-023-04051-9 (PMC10193804; doi:10.1186/s12887-023-04051-9)
Supplement: Supplementary file 1 — Supplementary Material 1 [file 12887_2023_4051_MOESM1_ESM.docx]

**Supplementary Table 1: Maternal awareness and knowledge of SIDS (perspectives on SIDS)**

| **Theme** | **Sub-themes with illustrative quotes** |
| --- | --- |
| **Awareness and Knowledge of SIDS** | ***Aware SIDS-like deaths in the community***   - *“My sister in-law had put the child to bed there after they just found the child dead” (P1, Chawama 2)* - *“According to what I read it was due to suffocation they slept at night so to my thought I think the mother was breast feeding then she fell asleep then the breast covered the nose of the baby causing suffocation that is what I thought” (P1, Chilenje-3)* - *“I just heard around but have never seen. I just heard maybe that the person had a baby then the baby just died while sleeping, I just heard but I don’t know anyone. Some people said maybe it is time, you can’t really specify, maybe the baby was sick because the baby was playing the baby cannot be poisoned maybe it was God”. (P4, Chilenje-2)*   ***Presumed cause of SIDS-like deaths***  *Smothering or overlay:*   - *“We hear them that the baby has died from home, maybe they have slept on the baby, others they say they just found them dead” (P4, Chawama-2)* - *“I just heard but I have never seen there was a woman……. when they went to sleep the baby was just okay and was 1 week old, I don’t know if she slept on the baby only to wake up to find the baby dead, then she called her parents that come and see what has happened in the bedroom. But I just heard, a friend of mine narrated it to me” (P1, Chilenje-1)*   *Soft-bedding suffocation:*   - *“Sometimes you may cover the baby in blankets they may suffocate, sometimes they have hiccup, or maybe they have not slept well, that may cause death” (P5, Chawama-1* - *“there is you find that you cover them [with blankets] then they have rolled over it covers their head, of course it may cause suffocation” (P3, Chawama-2)*   *Positional suffocation:*   - *“I just heard that the child died in sleep maybe was not feeling well they don’t talk or maybe the position they slept in” (P5, Chilenje-1)*   *Uncertain or Unexplained*   - *“I heard my neighbor had a 3 month old baby, they woke up in the morning the baby was okay after breast feeding the baby slept the mother went to wash out side then when she went back inside just found the baby was dead she didn’t know how the baby died only found the baby was not breathing” (P3, Chilenje-2)* - *“……what causes this sometimes is, you find you are busy working outside then you leave the child on the bed, you just go to find she is dead, what has killed him you don’t know” (P3, Chawama-1)*   *Baby abandonment:*   - *“like us where we stay one was pregnant she took medicine gave birth and threw the child in the toilet” (P4, Chawama-1)* - *“I have never had a child die, but I heard they say a lot that they have found a child is dead because they dump children in toilets others die in the house” (P2, Chawama-2)* - *“Others they dump children because they don’t love them or even putting in a plastic because they don’t love the baby” (P3, Chilenje-1)*   *Sick child:*   - *“I heard from my neighbor the child it’s like had (inaudible) they took him at the hospital he recovered then they brought him home it seems had another attack in the night in the morning was found dead” (P5, Chawama-1)* - *“I heard of it the child was sick she slept then when waking up her she thought that is just how the baby is unwell then prepared porridge now to go and carry the baby only to find the baby is dead” (P6, Chawama-3)*   *Fall from bed:*   - *“Sometimes we leave them on the bed while these children we have crawl, they may fall from the bed they die.” (P2, Chawama-1)*   *Milk aspiration:*   - *“What I can say is that babies you can’t know what can happen you find that maybe you as a mother you want to sleep then the baby was breast feeding….. then you sleep that one also causes the milk to go where it is not supposed to go then you just find the baby dead” (P2, Chilenje-3)*   ***Strategies to prevent SIDS***   - *“this is my first born so what I do is when we sleep where the baby is facing that is where I face too (everyone laughs) I don’t know what might happen” (P2, Chilenje-1)* - *“you can’t leave the child for a long time you have to leave the child with someone even when sleeping at least there has to be someone, its either I leave the child with the aunty or the grandmother” (P5, Chawama-3)* - *“I think we should wake up at night, especially the young ones because they wake up in the middle of the night to suck, so if you notice that from the time you slept the baby hasn’t woken up you should worry. With me if I reach 01 without noticing anything I wake up to check the pulse or the heartbeat then I lift him up, if she moves then I know she is okay. I also check for the sleeping position, if she turns then I know she is okay” (P5, Chilenje-3)* |

**Supplementary Table 2: Maternal perspectives on sleep practices**

| **Themes** | **Sub-themes with Illustrative Quotes** |
| --- | --- |
| **Sleep position** | ***Reason for chosen sleep position***  *Prevent aspiration:*   - *“At times after breast feeding when the baby faces up the milk will come out as vomit then it might enter the nose the baby won’t breathe well. At times when they sleep like this [supine], they don’t breathe well so for the sides its better because when they vomit it drops on the sides” (P4, Chawama 3)* - *“I put my baby to sleep on his sides because I think it’s safe even when the baby sleeps and vomits it’s easier for them to come out than when you put for the back it won’t even come out it will lead to choking” (P5, Chilenje-3)* - *“Can be for the sides or for the stomach it is the same because when the baby faces upright maybe was from breast feeding the milk might come out through the nose which is not good for the baby so position is also important can either be for the sides or stomach” (P5, Chilenje-1)*   *Infant breathes well:*   - *“it’s safe for me when it comes to rolling rather than making him to sleep with the stomach facing down, he might place the nose and fail to breath” (P2, Chawama-2)* - *“…. so that it is easy for him to breathe” (P4, Chilenje-3)*   *Infant Sleeps well:*   - *“Me the position is that for the sides (baby cries) (inaudible) children differ especially mine when I put him the other, they don’t sleep well but for the sides they sleep well” (P4, Chawama-1)*   ***Information sources***  *Family:*   - *“I: who do you listen to for advice on how to place your baby to sleep*   *P: our parents because we were once babies, so they told us how to keep the baby” (P4, Chawama-2)*   - *“When she was still a baby like when she was born I had my mother and my sister in-law but now as she is growing it’s me and my husband so we have noticed that now she is able to do this and that so I think we are also able to say this is better” (P1, Chilenje-3)*   *Healthcare workers:*   - *“I: who do you listen to when they tell you the position for placing the baby to sleep*   *P: just here at the clinic they teach us” (P2, Chawama-3)*   - *“I: who do you listen to on the position of placing the baby to sleep*   *P: the nurses at the hospital when they teach us” (P6, Chawama-3)* |
| **Bedsharing and room sharing** | ***Reasons for bedsharing or room sharing***  *Easy to monitor infant:*   - *“I sleep with my child because I have to sleep with my child, maybe at night the blanket covers the baby, so I have to be checking on the baby, so I have to sleep with the baby even when wants to feed I just carry her and start breast feeding perhaps it becomes cold I cover her” (P5, Chawama-3)* - *“I share the same bed and it is because it is easier for me to check the breathing, to feed him, to turn him when he is sleeping than him sleeping in another room” (P4, Chilenje-3)* - *“Maybe the child you put them to bed they are okay then at night the temperature rises so that is what we fear maybe they are sick they drop or maybe the thieves steal your baby or do something to the baby so unless when the child grows, he or she can be able to defend herself” (P5, Chilenje-1)*   *Convenient for breastfeeding:*   - *“If you have a child who is still an infant you have to sleep with them so that when they cry you breast feed them you can’t be managing to wake up you go and breast feed them. When you are sleeping it’s the same as being dead now if the child is near, we easily tell when the baby moves especially when they are 1 month, 10 months you should sleep with them on the same bed” (P1, Chawama-1)*   *Can’t afford separate room:*   - *“I sleep with my child on the same bed, same room here in Lusaka. Our stay is not good unless our friends from high-cost area who sleep some meters apart” (P1, Chawama-1)*   ***Strategies to prevent smothering***   - *“You just have to be conscious, ensure you see the position that he or she is sleeping. For me, I make sure that I put the baby where she can’t fall and me as a mother am careful I can’t sleep on her” (P, Chawama-1)* - *“The mind is alert we know that there is the baby here when the father is this side you know that the baby is on the middle so when turning you are careful the mind is always alert” (P7, Chilenje-2)* - *“… yes, especially when the baby is growing, they like touching things, making movements, so you just have to be careful, you even put pillows to prevent the baby from falling” (P5, Chilenje-1)* |
| **Sleep surface** | ***Preference for soft sleep surface***   - *“We need to spread where the baby sleeps so that it can be soft” (P1, Chawama-2)* - *“It has to be soft so that the baby would sleep comfortably” (P5, Chawama-3)* - *“It should be soft because you can’t put the baby where it is hard” (P2, Chawama-3)*   ***Blankets on sleep surface***   - *“we just fold a blanket for the child we lay him down and get another blanket we put for them to put the head so that they sleep well” (P4, Chawama-1)* - *“On the mattress that I lay on I use her blanket because it is a little thick that is what I use to put down” (P1, Chilenje-3)* - *“As for me even on the bed with a big mattress I get this baby blanket I fold it then I spread where they sleep, same in the village where you don’t have a bed you sleep on the ground, we get a blanket we spread for the baby” (P5, Chawama-1)* |
| **Bundling with blankets** | ***Reason for bundling with blankets***   - *“I was saying that the child has his blanket first, you start with their blanket then you put your blanket on top to keep them warm, so we start with their blanket and put ours, then we also join them to keep them warm” (P2, Chawama-1)* - *“.. yes, we use blankets……to avoid them from freezing” (P5, Chawama-2)* - *“I: do you use a blanket I have seen you have a shawl*   *P: yes, to prevent them from feeling cold” (P1, Chawama-2)* |

**Supplementary Table 3: Maternal perspectives on parental alcohol or tobacco smoking and breastfeeding**

| **Themes** | **Sub-themes and illustrative Quotes** |
| --- | --- |
| **Parental alcohol use** | ***Reasons for alcohol intake***  *Pregnancy related craving for alcohol:*   - *“alcohol I took on my first born child what causes that is the pregnancy takes us differently others when they are pregnant they just want to eat fruits, sour, cold tea others sugar solution, though cold things they don’t allow at the clinic even beer its cold that is why they don’t allow, it gives a problem to the child’s skin others brain so beer they don’t allow now the problem us who are pregnant we have that thirsty so mean how I used to drink I was mixing milk, sugar with chibuku shake shake I put in the jar I used to take a day 1 cup when I feel thirsty not every day” (P1, Chawama-1)* - *“Yes, they do depending on the appetite they have, you find sometimes the baby wants them to drink beer. When they drink that is when they feel better……. I think because pregnancy comes in different ways, others they will be craving for this and that….” (P2, Chilenje-1)* - *“.. yes, even my sister in-law, my co-sister in-law, she was craving but not every day but when she feels like then she drinks” (P3, Chawama-2)*   *Desire to have a beautiful child:*   - *“I saw someone drink who was pregnant. Some they drink shake shake to give birth to a beautiful child…... they are many” (P5, Chawama-1)*   *To cope with problems:*   - *“There are many who drink beer, they even smoke nsunko, because most women think that when they have a problem it will end with beer but by the time it is morning the problem will come back. It is better you remain sober so that you ask someone who can give you an idea if it’s a business you start beer is bad but, on my side, I don’t drink beer” (P1, Chilenje-3)*   ***Other women drink***   - *“I saw someone drink who was pregnant” (P5, Chawama-1)* - *“I don’t drink but I see people drink like the ladies from the bars you find that they drink even when they are pregnant” (P4, Chawama-2)* - *“They are many who drink even with heavy pregnancy just starting from day one until they give birth they are with beer” (P6, Chilenje-3)* - *“Yes, I have a neighbor we both where pregnant mine was small but hers was big, but she was drinking beer” (P3, Chawama-3)*   ***Aware of harms of alcohol on infant***   - *“I don’t drink but I see people drink like the ladies from the bars you find that they drink even when they are pregnant so you find those things are toxic and they affect the baby inside that may cause the baby to die in the womb, so alcohol is dangerous for our unborn babies” (P4, Chawama-2)* - *“I will just add on what R5 left, when we are pregnant at the clinic, they give us drugs they give us for blood appetite now if there is alcohol the drugs won’t be working so your health and that of the baby will be affected so in the future it will result into a problem” (P1, Chawama-3)* - *“So, you should not drink during pregnancy it can cause a lot of problems not only that even after the baby is born, we heard it can cause brain damage, there are so many things it can do to the baby” (P5, Chilenje-3)* - *“I also know the effects of drinking beer while you are pregnant so I was reading something, the most dangerous stage for drinking beer while you are pregnant is from 0 to 3 months, that is when the baby is forming the brain, but you can drink not just excessive” (P4, Chilenje-2)* |
| **Parental smoking** | ***Aware of harms of tobacco***   - *“Smoking is bad with or without pregnancy its bad because it destroys in the body” (P3, Chawama-1)* - *“Smoking is bad it destroys the lungs, it causes cancer it just causes a lot of diseases” (P5, Chawama-1)* - *“Cigarette is harmful to everyone they are times when someone used to smoke while pregnant but gave a normal birth then you also want to try then things don’t go well perhaps your immune system is not that strong it causes the child to die in the womb or after birth” (P6, Chilenje-3)*   ***Reason for using other tobacco products (nsunko)***   - *“I had a best friend who used to smoke nsunko putting it in the nose, and mouth but she was pregnant. We advised her that nsunko was not for pregnancy. Even at the clinic when they know, they will give you a form to go to the police because any problem they will blame the doctor, that they didn’t attend to the child while it is the mother who brought that problem a pregnant woman does not need to smoke those things are harmful” (P1, Chawama-1)* - *“Okay let me help her mostly if you have ever heard that a lady should be warm so there are substances that women take, though others say it is faster than anything else, some puts in the mouth and nose although some put in the vagina that it keeps warm and tightens. I think none of the types is good because if it has the capacity to burn you what more down there what can it do, imagine you are pregnant, you put that stuff down there it will go into your system and then it finds where the baby is so it can be harmful to the baby” (P5, Chilenje-3)*   ***Other women smoke***   - *“Me I can’t lie I have never smoked but I saw a lot of people who smoke just two days ago I saw one who is 15 years was smoking and drinking beer and the mother used to sale kachasu (Local win) so that boy has swollen legs” (P2, Chawama-1)* - *“I have never smoked unless my sister she smokes even when pregnant” (P3, Chawama-1)* |
